# Decrease in pH destabilizes individual vault nanocages by weakening the inter-protein lateral interaction

**DOI:** 10.1038/srep34143

**Published:** 2016-10-14

**Authors:** Aida Llauró, Pablo Guerra, Ravi Kant, Brian Bothner, Núria Verdaguer, Pedro J. de Pablo

**Affiliations:** 1 Departamento de Física de la Materia Condensada, UAM, Francisco Tomás y Valiente 7, 28049-Madrid, Spain; 2 Structural Biology Unit, Institut de Biologia Molecular de Barcelona, CSIC. Baldiri I Reixac 10, 08028-Barcelona, Spain; 3 Department of Chemistry and Biochemistry, Montana State University, Bozeman, Montana, USA; 4 Condensed Matter Physics Center IFIMAC UAM, Francisco Tomás y Valiente 7, 28049-Madrid, Spain

## Abstract

Vault particles are naturally occurring proteinaceous cages with promising application as molecular containers. The use of vaults as functional transporters requires a profound understanding of their structural stability to guarantee the protection and controlled payload delivery. Previous results performed with bulk techniques or at non-physiological conditions have suggested pH as a parameter to control vault dynamics. Here we use Atomic Force Microscopy (AFM) to monitor the structural evolution of individual vault particles while changing the pH in real time. Our experiments show that decreasing the pH of the solution destabilize the barrel region, the central part of vault particles, and leads to the aggregation of the cages. Additional analyses using Quartz-Crystal Microbalance (QCM) and Differential Scanning Fluorimetry (DSF) are consistent with our single molecule AFM experiments. The observed topographical defects suggest that low pH weakens the bonds between adjacent proteins. We hypothesize that the observed effects are related to the strong polar character of the protein-protein lateral interactions. Overall, our study unveils the mechanism for the influence of a biologically relevant range of pHs on the stability and dynamics of vault particles.

Vault particles are nanosized protein cages implicated in numerous cellular processes, including multidrug resistance, innate immunity and cellular transport^1^,^2^. However, the specific functions ascribed to this unique cellular organelle have not yet been irrevocably defined. Highly conserved and present in nearly all eukaryotes, vault particles consist of 78 copies of the major vault protein (MVP), which forms the MVP shell, plus three minor components. The less abundant species are the 193 kDa vault poly-ADP-ribose polymerase (VPARP), a 290 kDa telomerase associated protein 1 (TEP1), and several small non-coding RNA molecules (vRNA)^3^,^4^,^5^. A 3.5 Å resolution structural model for the rat vault assembly, based on X-ray crystallography, shows that the vault shell is structurally divided into identical halves, each one consisting of 39 copies of MVP^6^. A combination of hydrophobic and electrostatic interactions stabilizes the association of the two half-vault moieties. The entire particle forms an ovoid structure with overall dimensions of ∼40 × 40 × 67 nm^3^. Each MVP chain folds into 12 domains: a cap-helix domain, a shoulder domain and nine structural repeat domains that form the barrel (Fig. 1A)^6^,^7^. The strongest MVP-MVP lateral contacts are found between cap-helix domains, where hydrophobic residues stabilize the interface between helices on adjacent proteins.

Recombinant vaults can assemble *in vitro* after expression of MVP in insect cells. These vault-like structures are identical in size to natural vaults but have a hollow internal compartment that permits the storage of protein cargoes^8^. The ability to store hundreds of proteins, inherent biocompatibility and non-immunogenic cell response, make vault-like particles promising candidates as drug delivery vehicles for biomedical applications^1^. Indeed, the shell of recombinant vaults has been genetically modified to target packaging of specific payloads^9^,^10^,^11^ and cell specific targeting has been achieved by modification of the C- and N-termini of MVP^12^,^13^,^14^. Despite all these advances, however, little is known about the determinants that govern the structural dynamics of vaults, which is a fundamental step towards their use as artificial molecular transporters. The structural stability of vault particles has been studied across a range of pHs (3 to 8) and temperatures (4 to 70 °C), which revealed a variety of structures: full assemblies, half-vaults, states of aggregation and losses of secondary and tertiary structure^15^. To utilize vaults as containers it would be convenient to find an external parameter to control vault dynamics. For instance, during internalization of vault particles into cells through endocytosis, the acidic pH of the endosomal compartment might trigger vault dissociation^13^,^14^. Previous studies indicated that vault particles dissociate into halves at low pH, suggesting that this opening mechanism could be a way whereby vaults could deliver their cargoes^16^,^17^. However, an independent set of experiments using fluorescently labeled protein showed that recombinant vault particles are capable of half-vault exchange *in vivo* at neutral pH^18^, posing new question about what factors regulated vault dynamics and whether or not the pH was responsible for vault opening.

To investigate the impact of pH variation on vault particle stability we carried out experiments using three different techniques: Atomic Force Microscopy (AFM), Quartz Crystal Microbalance with Dissipation (QCM-D) and Differential Scanning Fluorimetry (DSF). Specifically, the environmental control of the buffer conditions at the AFM liquid chamber offers the possibility of studying structural changes of individual protein assemblies *in situ*^19^,^20^,^21^. Here we monitor the structural change of individual vault particles as pH varies from 7.5 to 5.2 in real time. Our results demonstrate that low pH mainly degrades the barrel zone inducing cracks that are not compatible with vault half opening^16^,^17^, but with the weakening of the inter-protein lateral interactions.

## Experimental Section

### Recombinant Baculoviruses

The generation of a recombinant baculovirus (rBV) containing the full length MVP was performed as follows: A DNA fragment containing the MVP sequence, flanked by NcoI and Kpnl restriction sites, was generated by PCR. The DNA fragment was digested with NcoI and KpnI and inserted into the multiple cloning site of the baculovirus transfer vector pFastBacHta (Invitrogen), previously digested with the same restriction enzymes. The resulting plasmid, pFB_MVP, was subjected to nucleotide sequencing to assess the correctness of the inserted MVP sequence, and was then used to produce the corresponding rBV using the Bac-to-Bac system and following the manufacturer’s instructions (Invitrogen)^TM^.

### Production and Purification of recombinant vaults

HighFive cells (Invitrogen)^TM^ were infected with rBVs at a multiplicity of infection of 5 PFU/cell. Cells were harvested at 48 hr post infection, washed using Phosphate Buffered Saline and pelleted with a 5 minute centrifugation at 3000 rpm. This pellet was re-suspended in 6 ml of buffer A (75 mM NaCl, 50 mM Tris pH 7.5, 1.5 mM MgCl_2_, 1 mM DTT) plus 1% NP-40 and protease inhibitors (Protease inhibitor cocktail tablets; Roche) and maintained on ice for 30 min. The re-suspended pellet was sonicated and cellular debris was removed by centrifugation at 10,000 rpm for 30 minutes. The supernatant was applied to 4 ml of buffer A with 25% sucrose and centrifuged at 37,000 rpm (using a SW41Ti rotor) for 2:30 hours. The resulting pellet was re-suspended in 600 μl of buffer A and centrifuged for 1 minute at 13,000 rpm. The supernatant was applied to a 25–50% sucrose gradient in buffer A and centrifuged for 45 minutes at 40,000 rpm. The gradient was fractionated and then analyzed by SDS-PAGE and negative-stain electron microscopy. Finally, fractions enriched in recombinant vaults were concentrated to approximately ∼5 mg/ml using a centrifugal filter device (Centricon YM-100; Millipore).

### AFM

AFM measurements were performed with a Nanotec microscope (Nanotech Electrónica S.L., Madrid, Spain) operated in Jumping mode plus^22^. In this mode, a force-vs.-distance curve is performed at every pixel of the image while the feedback is engaged at the bending signal (normal force). For moving from point to point, the tip retracts and moves laterally while being far from the surface, reducing the lateral forces that might damage the sample. Imaging force was maintained below ∼100 pN for all the experiments using rectangular silicon-nitride cantilevers (Olympus, RC800PSA) with a nominal spring constant of 0.05 N/m.

All the experiments were performed in liquid environment, with buffers at three different pHs: 7.5 (50 mMTris), 6.0 (50 mM Hepes) and 5.2 (50 mMNaOAc). All the buffers also contained 75 mM NaCl and 0.75 mM MgCl_2_. To perform the AFM experiments three different adsorption strategies were tested: (1) pre-incubation of the sample in the test tube under a specific pH conditions for 5 minutes prior to adsorption, (2) adsorption of the sample at pH = 7.5 for 30 minutes and posterior pH exchange of the buffer prior to imaging, and (3) adsorption of the sample at pH 7.5 followed by real time imaging of the sample while lowering the pH of the liquid cell buffer to 5.2. For all cases 1 μl of stock solution was diluted into 19 μl of the corresponding buffer. This 20 μl drop of diluted solution was incubated on a fresh highly ordered pyrolytic graphite (HOPG) surface (ZYA quality NT-MDT) and, after 30 minutes, washed with the desired buffer until a volume of 70 μl was reached. The tip was also pre-wetted with a 20 μl drop of buffer.

To exchange the buffer in real time a home-made system composed of two syringe pumps (NE-1000, New Era Pump Systems, Inc.) was coupled to the AFM^23^. Two experiments at different infuse and withdraw rates were performed (Fig. S3-B and -C). Both experiments started with an initial volume of 90 μl at pH = 7.5. To decrease the pH the initial volume was exchanged by infusing buffer at pH = 5.2. Slow exchange: at t = 0 min (V_o_ =90 μl) infusing was set at 3 μl/min (V_t = __18 min_ = 540 μl) and increased to 4 μl/min at t = 18 min, which was kept until finishing the experiment. Withdrawing of the buffer from the AFM liquid chamber started at time 38 min (V_t = __38 min_ = 134 μl) at a rate of 50 μl/min. The experiment was stopped at time 95 min, when the structures appeared totally collapsed (supplementary movie). We used a pH indicator to corroborate that the pH of the solution was 5.2 at the end of the experiment (time = 95 minutes). In the fast exchange experiment the height of the particles started to decrease at time 25 min (Figure S3B,C). At t = 0 min (V_o_ = 90 μl), infusing of buffer at pH 5.2 started at a rate of 20 μl/min and increased to 25 μl/min at t = 15 min. Withdrawing started at t = 13 min at 20 μl/min (V_t = __13 min_ = 350 μl). The experiment was stopped after 70 minutes, and a pH indicator was used to check that the pH of the solution was 5.2. The control experiment was performed by imaging an image of similar size (600 × 600 nm^2^) at constant pH (Figure S3A,C). After 24 images (t = 105 min) the height of the particle was only reduced a 2%. For comparison, in the fast exchange experiment after 15 images (t = 64 min) the height was reduced a 37% and in the slow exchange experiment after 24 images it was reduced to 25%.

All the images were acquired and processed with WSxM^24^. The height of the particles was considered as the maximum value obtained in the topographical profile. Distinct lateral dimensions of full- and half-vault particles allowed resolution of their structure in AFM images^25^. Full particles appeared as barrel-shaped structures whereas half-vaults presented a circular contour with a higher protrusion, corresponding to the cap, in the middle. All the AFM images had 128 × 128 pixels with an area <650 nm × 650 nm, which was enough to resolve the particles.

### QCM-D experiment

The viscoelastic changes associated with vault particles at different pHs (7.0, 6.0, 5.0, and 4.0) were studied by QCM-D (Q-Sense D300 system, Q-Sense AB, Goteborg, Sweden). For these measurements, gold quartz crystals (AT cut) with a fundamental resonant frequency of 5 MHz were used. Before loading of vault particles, control experiments using buffer changes (7.5, 6.0, 5.0, and 4.0) were conducted to quantitate the effect of buffers on frequency and dissipation. For the measurements, a baseline was established with 50 m MTris buffer (pH 7.5) and recorded for 10 minutes. After observing a stable base line, vault particles (50 μg in 600 μl Tris buffer, pH = 7.5) were gradually deposited on the crystal and allowed it to stabilize for 50 minutes. To remove loosely bound material, the surface was washed with 0.6 ml of buffer at pH = 7.5 for 10 minutes. Buffer was then exchanged, consecutively, to lower the pH to 6.0, 5.0 and 4.0. Time between exchanging buffers was 20 minutes. The same experiment was also conducted with 10 μg of sample to ensure that there was no artifact to signal coming from particle aggregation or the formation of particle multilayers. After each run, gold surface was cleaned and regenerated by soaking the crystal in a 1:1:5 mixture of H_2_O_2_ (30%), NH_3_ (25%), and distilled water at 60 °C for 15 min followed by exposure to UV light for 10 min. Frequency and dissipation values were recorded at fundamental frequency 5 MHz and three overtones (15 MHz, 25 MHz and 35 MHz); temperature was 24.5 °C.

### Thermal Denaturation

Thermal stability of vault particles was studied using four pHs (pH = 7.5, 50 mM Tris; pH = 6.0, 50 mMHepes; pH = 5.0, NaOAc 50 mM; pH = 4.0, NaOAc). These buffers also contained 50 mM MgCl_2_ and 75 mM NaCl. Vault particles were diluted in each buffer to a final concentration of 0.2 mg/ml. To each sample, 2.5 μL of 1% Sypro-Orange dye (Invitrogen 140 Inc. S6651) was added to obtain the final reaction volume to 25 μL. The assays were conducted in a qPCR instrument (Corbett Research, RG-3000). A temperature ramp from 25 to 99 °C, increasing 1 degree per minute was used. Lysozyme was run as a positive control at concentrations of 0.3 and 0.5 mg/ml. Melting temperature of samples was determined using the first derivative (dF/dT) of the raw fluorescence data. All these experiments were conducted in three technical replicates.

## Results and Discussion

### In-solution characterization of vault particles

**Figure 1:**
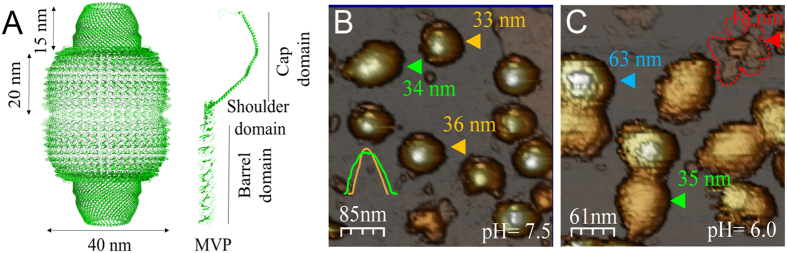
Vault structure. (**A**) Side view of a full-vault particle based on X-ray crystallography (PDB: 4V60)^6^ A full-vault consists of two identical moieties, each one comprising 39 major vault proteins (MVP). Each MVP is composed of twelve domains: nine structural repeat domains at the N-terminus (barrel domain), an α/β shoulder domain, a cap-helix domain and a cap-ring domain at the C-terminus. (**B**) General AFM topography image of vaults at pH 7.5. The images show two different configurations: reclined full-vault (green arrowhead) and half-vaults (yellow arrowheads). (Insets) Profile of the reclining full-vault (green) and half-vault (yellow). (**C**) General AFM topography image at pH 6.0 showing upright full-vaults (blue arrowhead), reclining full-particle (green arrowhead) and flower-like structures (red arrowhead). To guide the eye, the contour of the flower-like structure is shown with dotted red lines. Color scale bar: White-brown-grey, from the highest points to the substrate.

In AFM a tip with a radius of a few nanometers is located at the end of a microcantilever. The tip scans over the surface of biomolecular assemblies that have been previously adsorbed to a substrate in liquid milieu^26^. AFM images of vaults adsorbed on HOPG showed that at pH 7.5 full and half configurations were present (Fig. 1B). The lateral dimensions of the particles allowed us to resolve between full- and half-vaults (green and yellow profiles of Fig. 1B). If the solution pH was lowered from 7.5 to 6.0 before adsorbing the particles on the HOPG surface both configurations were also found. Figure 1C shows an AFM image with full particles at pH 6.0: either reclining on the barrel (green arrow) or standing up (blue arrow). Flower-like structures, consisting of a cap with the barrel domains open like petals, could be also seen under those conditions (red arrow)^27^.

We found no differences in the percentage of half and full configurations as a function of pH. However, particles at pH 6 decreased the average height by 14% and 7% for reclined full- and half-vaults, respectively (see Table S1 for a detailed description of statistics). This result suggests that while there is a similar dynamic equilibrium between the open and closed configuration regardless of the pH, the overall structure of the particle is weaker at lower pH. AFM images of particles incubated at pH 5.2 for 5 minutes before the adsorption on the substrate showed mostly empty areas and some clusters, suggesting high levels of protein aggregation under these conditions (data not shown).

### Imaging of adsorbed vault particles

**Figure 2:**
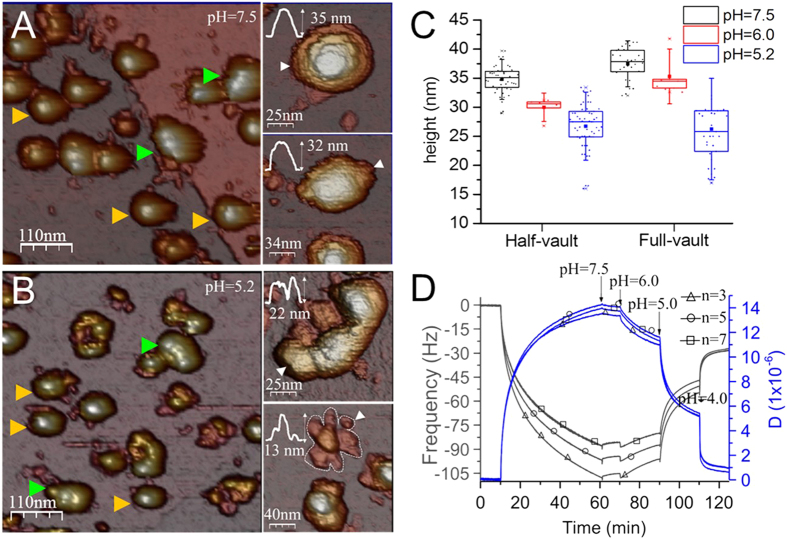
(**A**) General AFM topography of particles at pH 7.5. The image shows vaults with different configurations: reclined full-vault (green arrowheads) or half-vault (yellow arrowheads). High-resolution AFM images of vaults at pH 7.5 are shown on the right: half vault (top) and reclined full-vault (bottom). The white arrowheads indicate the line along which the height profile was taken. (**B**) AFM image of an area after lowering the pH to 5.2. Arrowheads indicate half-vaults (yellow) and reclined full-vaults (green). The images on the right show two typical vault structures found at pH 5.2: reclined particle (top), half-vaults and flower-like structures (bottom). Color scale bar: white-brown-purple, from the highest points to the substrate. (**C**) Box plot of the particles height at pH 7.5, 6.0 and 5.2. Both configurations (reclined full-vault and half-vault) show a progressive decrease in height. The error bar corresponds to the standard deviation, and the box range indicates percentile Q1 and Q3. The mean is indicated with a solid square. (**D**) QCM-D analysis of vault particles. Traces show the frequency in black (Y1 axis) and dissipation in blue (Y2 axis) for three overtones (n = 3, 5 and 7, corresponding to 15 MHz, 25 MHz and 35 MHz). After loading the particles on the gold-coated crystal the buffer was exchanged three times: from pH 7.5 to 6.0, 5.0 and 4.0 (black arrows). Evident changes in frequency and dissipation were observed every time that the pH was changed.

Because protein aggregation hampers the identification of the structures at single particle level, vault particles were anchored to the HOPG substrate at pH 7.5 and then the pH of the solution was decreased to 5.2 by washing the AFM liquid chamber, reassembling previous experiments performed by TEM^15^. In this situation, particles are not freely diffusing in solution and cannot form clusters, which allowed us to identify single vaults at pH 5.2. Imaging of the sample at pH 7.5 show intact particles easily identified as half- or full-vaults (Fig. 2A). In contrast, structures at pH 5.2 showed marked damage in the beta-sheet region of the barrel (Fig. 2B). Flower-like structure were also observed under these conditions (Fig. 2B, right bottom). For both cases (pH 7.5 and pH 5.2) the height of the flower-like structures (∼15 nm) corresponds to the size of intact cap region on native vaults (∼15 nm), indicating that the association between alpha-helices maintain their conformation even at acidic pH. The cap stability is believed to arise from the specific binding between the alpha-helix domains, which is a major contributor to interactions between MVPs proteins^6^. The strongest MVP-MVP contacts are found between cap-helix domains, where hydrophobic interactions dominate the packing (29 of the 41 pairs of residues forming the interface are hydrophobic). The intermolecular interactions in the barrel are weaker, mainly involving polar contacts (16 hydrogen bonds, 5 ionic bonds, and 10 hydrophobic bonds). Our AFM images show that at pH 5.2 the barrel region is damaged, likely due to the disruption of the polar residues at this central region (Fig. 2B, right top).

 The average height of particles decreased with pH as measured at 7.5, 6.0, and pH 5.2 (Fig. 2C and Table S1). At pH 5.2 particles present clear damage (Fig. 2B), so it seems reasonable to argue that at pH 5.2 the decrease in height was a consequence of the loss of protein subunits. However, at pH 6 the overall structure was preserved (Fig. 1C). In those cases, the decrease in height could be caused by the loss of proteins and/or by an overall softening of the structure, which at the same imaging force would result in a lower height.

### Rigidity and stability of vaults

The structural transition as a consequence of decreasing the pH was also studied by QCM-D^28^,^29^,^30^, which is a sensitive and straight-forward technique that measures viscoelastic changes associated with proteins and protein complexes. We have used it previously to study the pH-dependent conformational change of the small RNA virus CCMV^31^. To measure and compare the rigidity of vault particles, they were loaded onto gold-coated quartz crystals and changes in frequency (Δf) and dissipation (ΔD) as a function of pH were recorded simultaneously at three overtones (n = 3, 5, 7) (Fig. 2D). Vault particles were allowed to adsorb for 50 minutes which resulted in a decrease in frequency and an increase in dissipation. Loosely bound vault particles were removed by washing the sample at pH 7.5 for 10 minutes. The stable signal during the wash (60–70 mins.) indicates strong adsorption of vault particles to the surface. Introduction of pH 6.0 buffer led to a small increase in frequency (12%) and decrease in dissipation (15%). A more significant decrease in frequency (45%) and decrease in dissipation (52%) was observed after adding the pH 5.0 buffer. The change in frequency is related to a change in mass on the chip which could be due to detachment of material from the surface, partial disassociation/compression of vault nanocages, or a reduction of the vault hydration shell. The decrease in dissipation indicates an increase in the rigidity. A soft material is easily deformed during oscillations, which leads to high dissipation, while a rigid material has low dissipation because it strongly couples to the crystal. The increase in rigidity observed here is likely due to the collapse of the structure at lower pH. Further decrease of pH (from 5.0 to 4.0) was accompanied by a sharp increase in frequency (30%) and decrease in dissipation (33%). These results show that lowering the pH has a progressive destabilizing effect on the structure of vaults, which is not compatible with the division of vault particles into halves. A previous QCM study used a single pH change, precluding a distinction between a one-step transition (full-vault/half-vault) or a progressive disassembly process^16^.

Altogether our experiments suggest that at pH 6.0 the majority of vault particles maintain their barrel-shaped structure, although the reduction in height measured by AFM indicates that some of the inter-monomeric contacts at the barrel may have been weakened due to the decrease in pH. Below pH 6, particles start to aggregate when they are in solution and our AFM and QCM-D experiments on adsorbed particles reveal major structural changes. We performed thermal denaturation experiments with Differential Scanning Fluorimetry (DSF) to further investigate the effect of pH on the stability of the particles (Figure S1)^32^,^33^. When pH was changed from 7.5 to 6.0, the melting temperature (T_m_) was reduced by only 1.5 °C. At pH 5, a significant difference of 13 °C was observed in T_m_. DSF at pH 4 showed that particles were unstable even before heating. This result supports our hypothesis that below pH 6.0, particle integrity is compromised, but above that value, particles maintain their overall structure^13^,^14^.

### *In singulo* real-time structural dynamics as a function of pH

**Figure 3:**
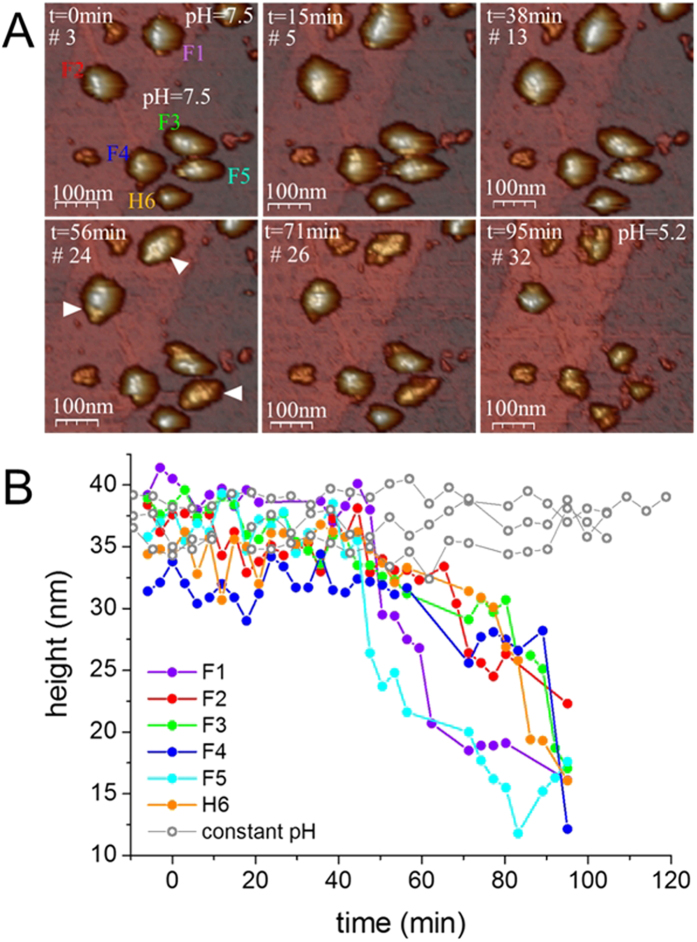
Real-time AFM images of individual vault particles while decreasing the pH from 7.5 to 5.2. (**A**) Different snapshots of the movie S1 showing the structural transition caused by the pH lowering. Each frame is labeled with the time and the corresponding number of frame. The pH was gradually decreased from pH 7.5 (frame #3) to pH 5.2 (frame #32). Frame #3 (t = 0 min) shows the initial configuration, with the particles labeled from 1 to 6: five reclined full-vault (F1-F5) and one half-vault (H6). White arrowheads (frame #24) indicate damage on the surface of the particles. At time 95 minutes (frame #32) the structures were collapsed. (**B**) Evolution of the height for each particle. From t = 50 mins onwards a steady decrease in height was observed. The grey lines are a control experiment and indicate the height evolution of a different set of particles that were imaged at constant pH under similar AFM imaging conditions (i.e., number of pixels, size of the image and imaging force).

Up until this point, our analysis has either used population averages to assess the properties of vault particles or caught individual frames through the pH-dependent destabilization process. To unequivocally identify the structural rearrangements occurring in individual vault particles we performed real-time AFM experiments while lowering the pH from 7.5 to 5.2. To accomplish this, the AFM liquid chamber was coupled to a two-syringe pump system that permitted *in situ* pH exchange. Briefly, one syringe flowed buffer at pH 5.2 into the AFM chamber where particles were adsorbed at pH 7.5, while the other syringe withdrew liquid. The experiment was stopped when the pH of the solution was 5.2 and the particles appeared collapsed (see Experimental Section for details). Before initiating the pH change, an area (∼700 nm^2^) containing multiple vaults was selected. This allowed single particles to be imaged in parallel. Figure 3A shows 6 selected frames of the area that was imaged 32 times while varying the pH. First, vault particles were equilibrated in pH 7.5 buffer. At t = 0 min (frame #3) we started the buffer exchange. The area was continuously imaged until the buffer was completely exchanged and the pH was 5.2 (t = 71 min, frame #26) (movie S1). Figure 3B shows the evolution of height of individual particles through the pH-exchange process (each curve (F1-H6) correspond to the particles labelled in frame #3). For example, curve F1 corresponds to the full-vault labeled as F1 and curve H6 to the half-vault labeled as H6. Analysis of the intermediate states showed that the height of the particles started to decrease at time ∼50 min. From this point onward, a monotonic decrease in height was observed. This decrease in height could be associated with the loss of structural integrity, including local damage on the barrel of the structure (white arrowheads, frame #24 of Fig. 3A).

**Figure 4:**
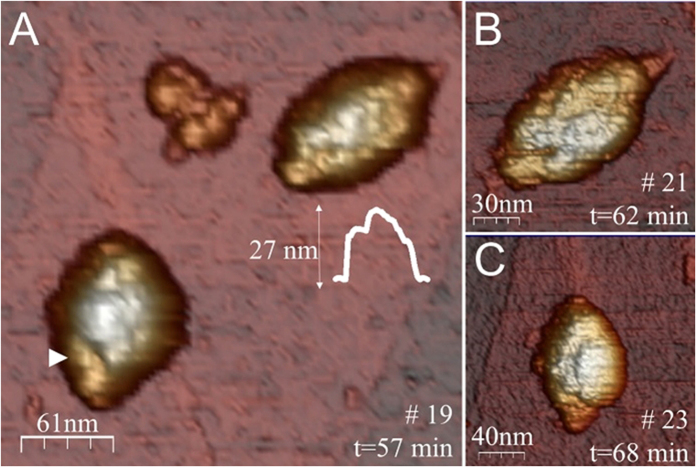
(**A**) High-resolution AFM images of F1 and F2 (see Fig. 3). The barrel zone present clear damage, suggesting the weakening of inter-monomeric contacts at the barrel. The white arrowhead indicates the damage of a region close to the shoulder domain. (Inset) Height profile of the particle on the top. (**B**) AFM image of the particles on the top of (**A**) at a later time. (**C**) AFM image of the particle at the bottom of (**A**) at a later time. Color scale bar: white-brown-purple, from the highest points to the substrate.

 Higher resolution images of F1 and F2 (Fig. 3A) acquired at t = 60 minutes confirmed that lowering the pH caused the formations of depressions on the top surface of the particles (Fig. 4). For instance, the particle on the top of Fig. 4A shows barrel structure debris with an average height of ∼27 nm (lower than the 35 nm of the native particles). A weakening of lateral bonds between neighboring MVPs might explain this bumpy surface and decrease in height. An image of the same particle at later time (Fig. 4B) shows a depression of the barrel zone. Likewise, the particle at the bottom of Fig. 4A presents a decrease in height close to the shoulder domain that connects the barrel with the cap region (white arrowhead). A later image of the particle (Fig. 4C) shows that the depression extended to the other half-vault moiety, forming a longitudinal crack. These data do not suggest that lowering the pH promotes the opening of vault particles into halves. It could be argued that the substrate would impair such opening of the vaults. The measured absence of collapse^25^ informs about a minimum vault-surface interaction. Consequently, this interaction locally affects a small area which just accounts for a few of the 39 MVP subunits joining the two moieties, even if assuming some deformation. Since vault particles are soft protein shells that can accommodate their shape to structural defects^25^, any equatorial division initiated at the top of the barrel would grow until reaching the bottom and would be evident in our AFM images. The substrate would avoid disjoining a few MVP-MVP bonds of the barrel bottom, but the rest would open because no surface holds them together. In addition, we found that full-particles laying on their cap maintain their two moieties at pH 5.2 (Figure S2). Noticeably, some of these structures decrease their height from 70 nm to 51 nm, which agrees with a weakening of the overall structure and not with a half-vault opening.

Finally, because the AFM probe establishes mechanical contact with vault particles during the imaging process^22^, it was important to rule out this effect as the main source of damage or weakening. To address this issue we performed control experiments by imaging vaults at neutral pH under the same AFM conditions: image area, set-point force and number of pixels. For example, we scanned vault particles 33 times during 110 minutes at constant pH 7.5 (Figure S3) and did not observe a significant decrease in height (grey curves, Figs 3B and S3C). In addition, we performed real time experiments with a faster pH exchange rate and observed that the onset of decrease in height started at an earlier time (Figure S3). In that case, the height of the particle started to decrease after 20 minutes, which is less than the 50 minutes required in the slow exchange experiment (Fig. 3). Therefore, our control experiments proved that the pH was the main responsible for vaults destabilization^16^,^17^.

### Biophysical implications and outlook for controlled payload delivery

**Figure 5:**
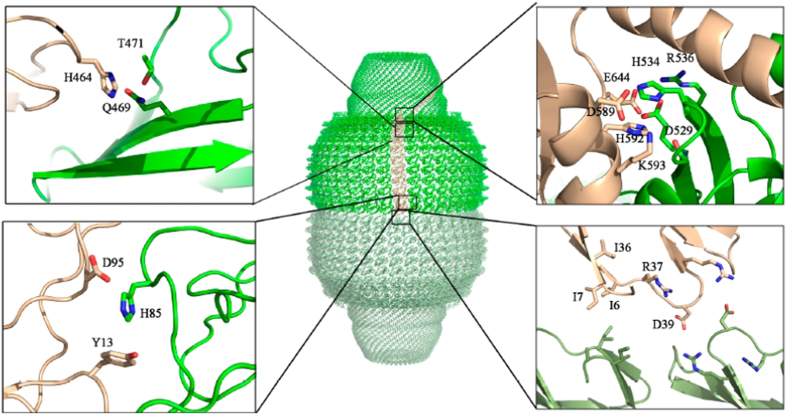
(**A**) Structure of the vault shell, colored in green and pale green for the top and bottom half-vault moieties, respectively. One of the 78 MVP copies forming the particle shown in brown (PDB id: 4JL8)^6^. The left insets show a close up of the MVP-MVP lateral electrostatic interactions mediated by histidines in the R2 (bottom) and R9 (top) domains. The top right inset shows the MVM-MVP lateral electrostatic interactions in the shoulder domain. The right bottom inset shows the R1-R1 interactions established at the half-vault interface.

The detailed analysis of the lateral inter-subunit interactions, using the refined structure of the particle (PDB id: 4HL8)^34^, evidenced the presence of a number residues at the MVP-MVP interfaces [His85 (R2), His464 (R9) and, His534 and His592 (shoulder domain)] that appear easily protonable at mild acidic pH (Fig. 5). We speculate that the protonation of these amino acids would destabilize the ionic interactions in the region, weakening of the lateral MVP-MVP contact interfaces.

The weakening of lateral interprotomeric interactions are also compatible with our previous mechanical study showing that AFM tip-induced mechanical fractures occur along lateral MVP-MVP contacts and not at the waist^25^. Another protein assembly that also displays longitudinal fractures and self-healing capabilities are microtubules(MT)^35^,^36^. In MT, this high reversibility of lateral bonds repair the cracks between shortening protofilaments in the MT wall and serve as means of inhibiting de-polymerization. It seems interesting that the spatial distribution of tubulin protofilaments forming MT resembles that of MVP monomers in the barrel zone, which also bundle parallel to one another and form a cylindrical structure. In our case, decreasing the pH from 7.5 to 5.2 leads to the weakening of the interactions on the vault barrel and further collapse of the structure. These gradual pH-dependent dynamics might be exploited during vault cellular uptake upon endocytosis, where the pH of the environment suffers mild acidification^13^,^14^.

## Conclusions

In this paper we have combined single particle and bulk techniques to study the structural dynamics of individual vault particles as a function of pH. Our results show that at pH 7.5 and 6.0 the quaternary structure of vaults(observed as full- or half-particles) was maintained. Although the overall structure was preserved, our AFM experiments indicate that at pH 6.0 the height of the particles was reduced a 15%. The adsorption of individual vaults on substrates allowed us to monitor the pH-dependent dynamics of single particle in real time. Our experiments showed that lowering the pH does not open vaults into halves, as suggested before^7^,^16^,^17^, but promote a global destabilization of the particle that is governed by the weakening of inter-monomeric contacts at the barrel and shoulder regions. Overall, our results reveal new insights into the pH-dependent dynamics of vault particles and might offer a means of controlling artificial cargo delivery upon cell endocytosis, where the pH of the environment gradually decreases.

## Additional Information

**How to cite this article**: Llauró, A. *et al*. Decrease in pH destabilizes individual vault nanocages by weakening the inter-protein lateral interaction. *Sci. Rep.* **6**, 34143; doi: 10.1038/srep34143 (2016).

## Supplementary Material

Supplementary Movie

Supplementary Information
