# Supplementary material for: Decrease in pH destabilizes individual vault nanocages by weakening the inter-protein lateral interaction
Source: Sci Rep. 2016 Oct 14;6:34143. doi: 10.1038/srep34143 (PMC5064368; doi:10.1038/srep34143)
Supplement: Supplementary Information [file srep34143-s2.pdf]

Supporting information

Decrease in pH destabilizes individual vault nanocages by weakening the inter-protein lateral interaction

Aida Llauró, Pablo Guerra,‡ Ravi Kant, Brian Bothner, Núria Verdaguer, Pedro J. de Pablo

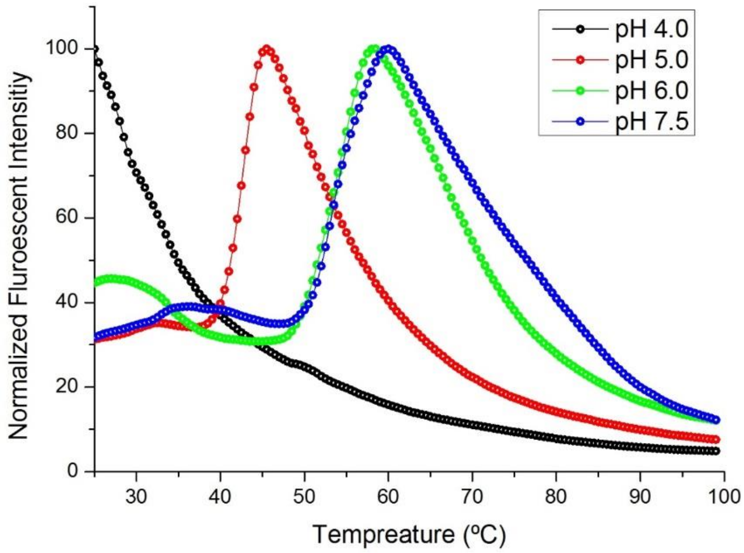

Figure S1. Differential scanning fluorescence at pH 7.5, 6.0, 5.0 and 4.0. The corresponding melting temperature are 60.5±0.5 ºC, 59±0.5 ºC and 46.0±0.5 ºC for pH 7.5, 6.0 and 5.4, respectively. Particles are not stable at pH 4.

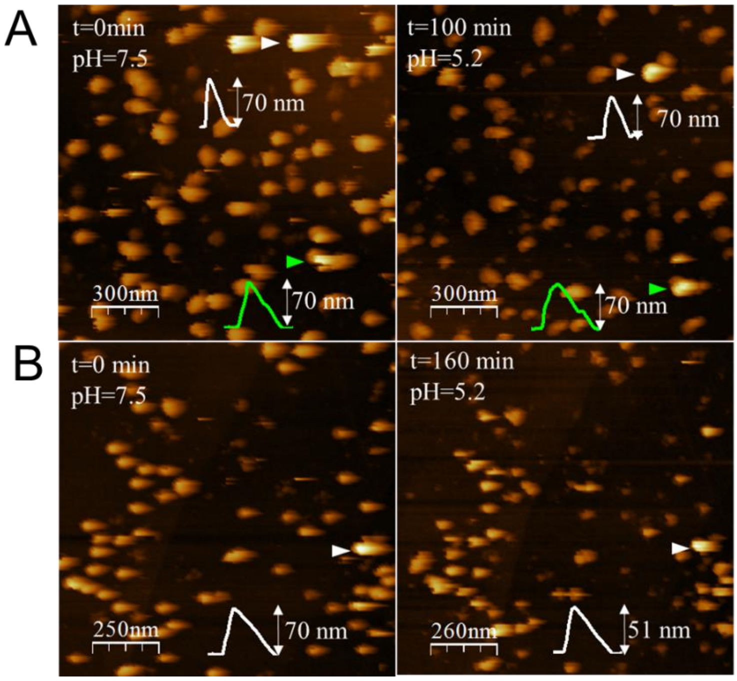

Figure S2. AFM topography evolution of single stand-up full-vault from pH 7.5 to 5.2. (A) AFM images of the same area before and after changing the pH from 7.5 to 5.2. At pH 5.2 some particles present a stand-up configuration, proving that lowering the pH not necessary open particles into halves (white and green arrows). (inset) Profiles of the particles. (B) AFM images of the same area before and after changing the pH from 7.5 to 5.2. (inset) Profile of the particle. pH lowering reduced the height of the particle likely as a consequence of an overall weakening of the structure. Color scale bar: white-golden-brown, from the highest points to the substrate.

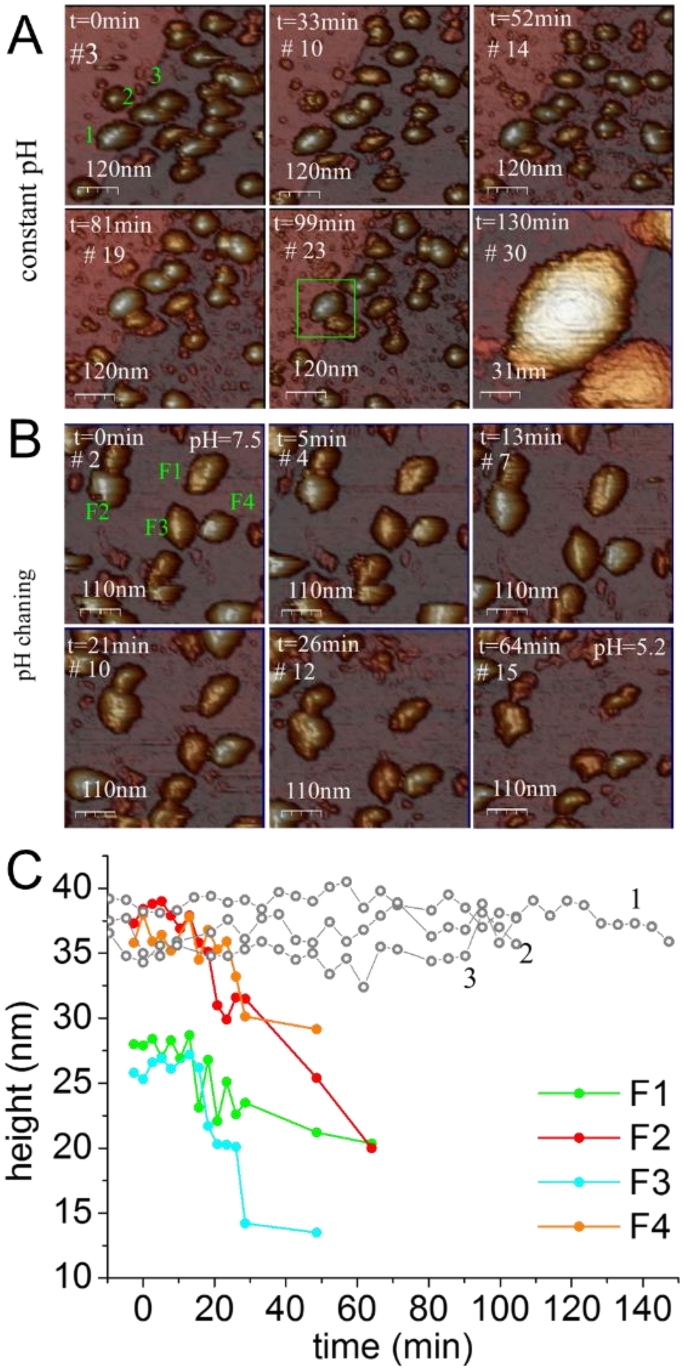

Figure S3. Control AFM experiments. (A) A region of vault particles (600x600 nm^2) imaged at constant pH for 105 minutes (24 frames). The figure shows different snapshots of the process, each frame is labeled with its corresponding time. Labels 1, 2 and 3 correspond to two reclining full-vaults and one half-vault, respectively. The evolution of their height is plotted in Figure S3-C (grey lines). From time 103 minutes onwards consecutively higher-resolution images of full particle 1 were taken (green square, frame #23). Even at this higher resolution (200x200 nm^2) the height of the particle was roughly maintained (grey line 1, Figure S3-C). (B) Evolution of the structural transition caused

by lowering the pH at a faster exchange rate (in comparsion with the experiments shown in Figure 3). Each frame is labeled with the corresponding time. Frame #1 (t=0 min) shows the intical configuraiton, with the particles labeled from F1 to F4 (all the particles were full-vaults). Particles F1 and F3 were likely damaged from the beginning, which caused a lower initial height. (C) Evolution of the height for the experiments shown in S3-A and S3-B.

	Full-vault		Half-vault	
	n	*height*±SD	*n*	*height*±SD
pH=7.5	30	37±3	47	35±3
pH=6.0	9	35±3	6	30±2
pH=5.2	18	25±6	35	27±4

Table S1. Height of the particle as a function of pH (mean ± standard deviation). Particles at pH 7.5 and 6.0 were incubated at the corresponding buffer before adsorption. Particles at pH=5.2 were adsorbed at pH 7.5 and then the pH of the solution was decreased to 5.2. This table contains information at three different pH, data at pH 5.2 comes from 6 different set of experiments, data at pH 7.5 comes from 7 different set of experiments (in both cases we found that the results were reproducible). Although the statistics at pH 6.0 contains data from 2 different sessions, in both occasions the results have been consistent with the rest of data. Shapiro-Wilk normality tests show that only data at pH 7.5 follows a normal distribution ($p<0.05$). Non-parametric tests show that the populations at the different pHs are significantly different ($p<0.05$). In addition, the agreement of these AFM results with our thermal denaturation and QCM-D support the fact that at pH 6.0 particles might have suffered some damage, although it is below pH 6.0 that the structure

is severely compromised (see Figure 2B (AFM), 2D (QCM-D) and S1 (thermal denaturation)).

Caption for supplementary movie.

This movie is composed of 32 frames, obtained during 77 minutes of elapsed time at a constant force of 150 pN. See a detailed description in the AFM section of the manuscript and the caption of figure 3.
